# Invasive Group A Streptococcal Disease in Nursing Homes, Minnesota, 1995–2006

**DOI:** 10.3201/eid1405.0704072

**Published:** 2008-05

**Authors:** Jean Rainbow, Brenda Jewell, Richard N. Danila, David Boxrud, Bernard Beall, Chris Van Beneden, Ruth Lynfield

**Affiliations:** * Minnesota Department of Health, St. Paul, Minnesota, USA; † Centers for Disease Control and Prevention, Atlanta, Georgia, USA

**Keywords:** Streptococcus pyogenes, nursing home, epidemiology, outbreaks, infection control, research

## Abstract

Nursing home residents are at high risk for invasive group A streptococcal (GAS) disease, and clusters of cases in nursing homes are common.To characterize the epidemiologic features of invasive GAS disease in nursing homes, we conducted active, statewide, population- and laboratory-based surveillance in Minnesota from April 1995 through 2006. Of 1,858 invasive GAS disease cases, 134 (7%) occurred in nursing home residents; 34 of these cases were identified as part of 13 clusters. Recognizing cases of GAS disease in nursing homes posed challenges. Measures to ensure identification of case-patients as residents of specific nursing homes need to be included in standard guidelines for the prevention and control of invasive GAS disease in this setting.

## Abstract 2

Nursing home residents are at high risk for invasive GAS disease; clusters are common.

*Streptococcus pyogenes,* or group A *Streptococcus* (GAS), is most commonly associated with noninvasive conditions such as pharyngitis and impetigo but can also cause severe invasive GAS infections such as necrotizing fasciitis and streptococcal toxic shock syndrome (STSS) (*1*–*3*). Risk factors for invasive GAS disease include advanced age, diabetes mellitus, cardiac disease, chronic obstructive pulmonary disease, cancer, immunocompromising conditions, and varicella (*4*,*5*). Most nursing home residents have at least one of these risk factors, which makes this population especially vulnerable to invasive GAS disease. An estimated 8,950 to 11,500 (3.5/100,000 population) invasive cases and 1,050 to 1,850 deaths occur in the United States annually (*6*). The incidence among persons >65 years of age of 9.4/100,000 population is almost 3 times that of the general population (*6*).

Most of the literature about GAS disease in nursing homes has focused on acute outbreaks with little attention paid to sporadic disease in this setting (*7*–*19*). Factors contributing to these outbreaks include GAS-infected caregivers, inadequate infection control measures, resident-to-resident spread, and the presence of a chronically infected or persistently colonized resident (*7*–*17*). On the basis of our experience with GAS surveillance in Minnesota, we suspect that a lack of recognition of GAS disease occurrence within nursing homes may also be a contributing factor.

We describe the occurrence of invasive GAS disease among residents of nursing homes in Minnesota over 11 years, the challenges we encountered with surveillance, and our efforts to prevent and control the spread of disease in this setting. Our findings will be useful in the development of guidelines for the prevention and control of GAS disease in nursing homes.

## Methods

### Surveillance

We began active, statewide, population- and laboratory-based surveillance for invasive GAS disease in April 1995 through Active Bacterial Core surveillance (ABCs), part of the Emerging Infections Program of the Centers for Disease Control and Prevention (CDC) (*20*). The population of Minnesota was 4.9 million in 2000. Invasive GAS disease is defined as GAS isolated from a normally sterile site such as blood or cerebrospinal fluid or from a wound when accompanied by STSS or necrotizing fasciitis (*20*). To ensure complete case capture, laboratories either submit computerized lists of all GAS-positive cultures from normally sterile sites at least monthly or are contacted twice monthly by Minnesota Department of Health (MDH) staff, and audits are completed routinely. Hospital infection control practitioners then complete standard report forms for cases of invasive disease, and all GAS isolates are sent to the MDH Public Health Laboratory. All GAS isolates undergo pulsed-field gel electrophoresis (PFGE) with *Sma*1 by methods described elsewhere, with the exception that an *Enterococcus* isolate was used as the standard (*21*). PFGE patterns are evaluated both visually and with BioNumerics software (Applied Maths, Kortrijk, Belgium) by using the dice coefficient. For patterns to be considered indistinguishable, they must visually appear identical and the DNA patterns must differ by <1.5% with respect to molecular weight. Isolates are also sent to CDC for *emm* typing (*22*).

Beginning in 1995, information about a case-patient’s residence was collected on the case report form, including street address, city, state, and ZIP code. In 1998, a question was added about whether the case-patient had been a resident of a nursing home, long-term care facility (LTCF), or other chronic care facility for at least 30 days before the date the culture was collected. Persons living in group homes, prisons, rehabilitation hospitals, or who were going to facilities for daily outpatient therapy were not included. Beginning in 2002, the name of the facility was collected. Addresses for case-patients >55 years of age were also checked by a reverse address directory to see whether they corresponded with that of an LTCF. Information about size, location, and classification of LTCFs was collected from a directory of Minnesota licensed, certified, and registered healthcare facilities.

For the purposes of this study, only case-patients who could be confirmed as residents of nursing homes were included because accurate denominator data were only available for this group. In Minnesota, a nursing home is defined as a facility that provides nursing care to >5 persons who are not in need of acute care facilities but require nursing supervision on an inpatient basis. Denominators for calculating incidence were derived from the 2000 US Census data as reported by the Minnesota State Demographic Center, which describe the population living in group quarters by age and type of quarters (*23*).

### Cluster Identification

We defined a nursing home cluster as >2 cases in residents of a nursing home in which isolates were nearly identical as determined by PFGE (PFGE patterns within a 3-band difference [*24*]) during a 12-month period. PFGE patterns were used for cluster identification because they were more discriminating than *emm* types (e.g., several PFGE patterns were typically found to correspond to 1 *emm* type, while PFGE patterns were not found to have multiple *emm* types), and PFGE was readily available in our laboratory. We chose 12 months because we observed that invasive cases with indistinguishable patterns sometimes occurred many months apart within a facility. Beginning in 1995, case reports were reviewed regularly by address, facility name, and PFGE pattern to look for clusters.

EpiInfo version 6.0 (www.cdc.gov/epiinfo/Epi6/EI6dnjp.htm) was used for statistical analysis. χ^2^ test was used to determine statistical significance of differences in proportions for discrete variables, and a *t* test was used to determine whether the difference in means was significant for continuous variables.

### Intervention

Since 1997, whenever a cluster was identified through ABCs, the nursing home was contacted by MDH and encouraged to conduct retrospective and enhanced prospective surveillance for invasive and noninvasive GAS infections. This surveillance included reviewing culture logs to identify noninvasive GAS infections and residents with chronic or recurrent infections and reviewing reported staff illnesses to identify a possible source of GAS. Clinical examples of possible GAS infections were provided so the facility could consider the possibility of earlier undiagnosed GAS disease and recognize new suspect cases. For prospective surveillance, the nursing home was encouraged to obtain cultures for any suspected infection and to ask laboratories to save GAS isolates for PFGE and *emm* typing. A follow-up letter and packet of information about GAS and infection control measures were also sent to the nursing home. We offered assistance of further investigation but most often had no further contact with the facility. In 2004, after noting that we seldom observed another case at a facility after we contacted them regarding a cluster, we began to contact a facility any time we received a report of a single case.

In Minnesota, MDH staff cannot conduct an investigation in a nursing home without an invitation from the facility. Only 2 facilities with clusters requested our assistance. These investigations have been described in detail elsewhere (*25*,*26*), but in both instances cultures were collected from residents and staff (all residents and staff at facility G; all but 1 resident who refused and 3 staff members in the affected unit at facility B). Those with positive GAS cultures were treated with antimicrobial agents (clindamycin at facility G and penicillin and rifampin at facility B). Formal infection control educational sessions were provided for staff on the same day that cultures were collected.

## Results

### Surveillance

**Figure 1:**
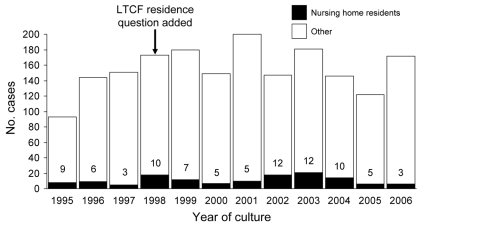
Annual number of cases of invasive group A streptococcal infections and percentage of cases occurring among nursing home residents, April 1995–2006. LTCF, long-term care facility.

**Figure 2:**
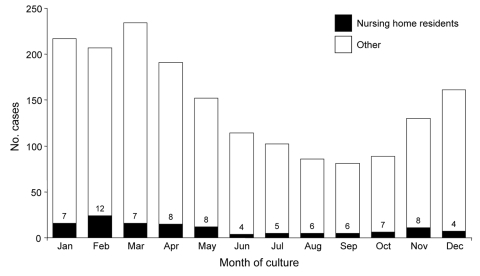
Number of cases of invasive group A streptococcal infections and percentage of cases occurring among nursing home residents by month of culture, Minnesota, 1995–2006.

From April 1995 through 2006, 1,858 cases of invasive GAS disease were reported among Minnesota residents; 642 (35%) were in persons >65 years of age. One hundred seventy-five case-patients were identified as LTCF residents on their case report forms. Twenty-three of these case-patients resided in non–nursing home settings such as assisted living or group homes, and we were unable to determine the type of setting for 18 of those designated as LTCF residents. One hundred thirty-four (7%) of our case-patients were known to be nursing home residents. The number and percentage of all cases associated with nursing homes fluctuated over time; from 6 to 21 cases were identified among nursing home residents annually, representing 3%–12% of all case-patients each year (Figure 1). Seasonal variation of invasive GAS infections was noted among both the general population and nursing home residents (Figure 2), with peak incidence in the winter and spring and little disease noted in late summer and early fall.

The age of nursing home case-patients ranged from 36 to 100 years of age (median 84 years); 58% were women, 87% had positive blood cultures, 36% had bacteremia without another focus of infection, 32% had cellulitis, and 12% had pneumonia. The case-fatality ratio of all case-patients with invasive GAS was 12%. Among case-patients >65 years of age, the case-fatality rate of nursing home resident case-patients (n = 121) was 35% compared with 18% of case-patients who were not nursing home residents (n = 521).

In 2000, 37,542 Minnesota residents >65 years of age lived in nursing homes, and 556,724 lived in their own homes or other group quarters. During 2000, the incidence of invasive GAS infections among Minnesota nursing home residents >65 years was 18.6 cases/100,000 population compared with 6.8 cases/100,000 among those >65 years who did not reside in nursing homes. Estimated annual incidence for nursing home residents >65 years varied from 13.3 cases/100,000 in 1997 to 50.6 cases/100,000 in 2003, while the estimated incidence for non–nursing home residents >65 years was 8.6 and 9.3 cases/100,000 during the same years.

*Emm* type was available for 117 (87%) of the nursing home case-patient isolates. Of 21 different *emm* types identified, 4 (*emm* 1 [21%], *emm* 89 [15%], *emm* 28 [13%], and *emm* 03 [11%]) accounted for 60% of the isolates. Among 1,416 (82%) non–nursing home case-patients, 5 *emm* types accounted for 62% of the isolates (*emm* 1 [24%], *emm* 28 [13%], *emm* 03 [11%], *emm* 12 [10%], and *emm* 89 [4%]). Although total numbers of cases varied considerably from one year to the next, the proportion of disease caused by the most common *emm* types fluctuated little.

### Cluster Identification

Of the 444 licensed nursing homes in Minnesota, 91 (20%) were known to have at least 1 case of invasive GAS disease during the study period. Sixty-seven (74%) of these facilities had a single case; 13 facilities had 2 cases; and 11 facilities had >3 cases. Of 24 facilities that had >2 cases, 13 (54%) met the definition of a cluster as previously defined (Table). We found that PFGE patterns for isolates from the same facilities were either indistinguishable from each other or distinctly different (>3 bands different).

**Table:** Clusters of invasive group A streptococcal disease in nursing homes, Minnesota, April 1995–2006

Facility	Year(s)	Month of onset for first case	No. cases	Interval between first and second case	Interval between first and last case	*emm* type
A	1995	Apr	4	1 mo	7 mo	01
B	1996–1997	Jan	5	10 mo	16 mo	89
C	1998	Feb	2	5 mo	5 mo	01
D	2000	May	2	2 mo	2 mo	82
E	2001	Apr	2	3 wk	3 wk	01
F	2002	Feb	2	9 mo	9 mo	28
G	2002	Mar	3	3 wk	3 mo	01
H	2002–2003	Dec	3	3 mo	3.5 mo	03
I	2003	Jan	2	2 mo	2 mo	89
J	2003	May	2	1 wk	1 wk	28
K	2003	Feb	3	2 wk	9 mo	01
L	2003–2004	Dec	2	1 mo	1 mo	12
M	2004	Feb	2	4 d	4 d	05

Four nursing homes that had clusters also had additional cases that did not fit the definition for inclusion in the cluster, either because a case isolate had a distinctly different PFGE pattern, a case did not occur within 1 year of the other cases, or both. One of these facilities had 4 such cases. In addition, 11 other nursing homes each had 2–3 cases that did not fit the definition of a cluster.

All but 2 clusters were caused by the most common *emm* types. *Emm* 1 was the most common cause of invasive disease (causing 24% of all cases) and also the cause of 5 (38%) clusters.

Eighteen of 21 pairs (86%) of consecutive cases occurring within 12 months of each other in the same facility had matching PFGE patterns, while 13 (93%) of 14 pairs of consecutive cases occurring within 3 months had matching patterns. The occurrence of a third case in a nursing home was not dependent on the first 2 case isolates having the same PFGE patterns; 6 (46%) of 13 facilities in which the first 2 case isolates had different PFGE patterns and 5 (45%) of 11 facilities in which the first 2 case isolates had matching PFGE patterns subsequently had more cases.

No significant difference was found for age, sex, or type of infection between cluster and sporadic cases. Forty-one percent of case-patients with cluster-associated cases died, compared with 32% of patients with sporadic cases, but this difference was not significant (p = 0.33).

### Intervention

In 32 (86%) of 37 encounters with nursing staff, the person contacted was not aware of a diagnosis of invasive GAS disease among their residents before our call. In addition, even when our contacts (usually either directors of nursing or nurses designated to oversee infection control for the facility) did know of the diagnosis, they generally had little knowledge about GAS disease. Before 2004, we noted that 9 of 12 nursing homes did not identify additional cases of invasive disease after our call. In 2004, we began notifying nursing homes after we identified single cases in their facilities. Since then, we are aware of only 1 facility with a cluster, and that facility had 2 cases 4 days apart.

We collected throat and skin lesion cultures from staff and residents for a unit with a cluster of invasive GAS disease at facility B and from staff and residents of the entire nursing home at facility G. At facility B, 2 (2.7%) of 75 throat cultures from staff and 2 (5.9%) of 34 throat cultures from residents were positive for GAS; 5 (6.2%) of 81 throat cultures from staff and 2 (4.5%) of 44 throat cultures of residents were positive for GAS at facility G. All of those with positive throat cultures were asymptomatic at the time of culture. All except 1 isolate from a staff person at facility G who did not provide direct patient care had PFGE patterns indistinguishable from those associated with the invasive cases at the facility. Those with positive cultures were each treated with a course of antimicrobial drugs, and no additional cases were detected at either facility.

## Discussion

Minnesota has had a unique opportunity to conduct active, population-based surveillance for invasive GAS disease for >11 consecutive years with nearly complete case reporting plus further molecular characterization of associated GAS isolates. Findings from this statewide surveillance, our review of the strengths and weaknesses of GAS surveillance specific for nursing homes, and further evaluation of factors associated with clusters of GAS in this setting provide information to aid in the development of effective national guidelines for the prevention and control of GAS infections in this vulnerable population.

Although <2% of Minnesota’s population resides in nursing homes, at least 7% of invasive GAS cases occurred among this population. As noted in other studies (*27*), we also found that the case-fatality rate was higher for nursing home residents than for the rest of the population. Much of this increase in illness is likely due to the frequency of risk factors for invasive GAS disease among this population (e.g., advanced age and underlying diseases such as diabetes and chronic obstructive pulmonary disease); however, the increase may also be due to difficulties of limiting the introduction and transmission of GAS in this or any institutional setting or in a closed population.

The true incidence of GAS disease in nursing homes and the occurrence of clusters are likely higher than detected by our surveillance system. Collection of specimens from febrile nursing home residents is limited when infections in nursing home residents are treated empirically. In addition, our early surveillance methods likely misclassified the residence of GAS case-patients. We found that the street addresses for patients that were obtained from hospital admission records were often not the addresses of the nursing homes where case-patients resided but were instead the home address of a spouse or other family member. The percentage of case-patients with invasive GAS disease identified as living in nursing homes rose markedly in 1998 when a specific question about LTCF residence was added to the ABCs case report form. In 2002, we began collecting the name of the facility where potential case-patients resided, enabling nursing home residence to be confirmed. Because of these improvements in methods over time, we cannot appropriately compare our early nursing home disease rates to those calculated from more recent data to draw conclusions about changes in trends.

Prevention and effective control of GAS infections in nursing home residents can be improved with changes in surveillance. Knowledge of the initial case in a facility may help prevent a second case through review and improvement of infection control in the facility, the identification of and treatment for a colonized or infected staff member, or segregation of infected patients. We found that nursing homes were frequently unaware that their hospitalized residents had invasive GAS disease until notified by public health officials. All GAS infections identified by referring hospitals must be reported back in a timely manner to the nursing homes from which a patient was transferred. Surveillance for noninvasive GAS infections may also be needed. Because these infections are not reportable, nursing home staff and public health personnel may not be aware of the first introduction of GAS into a facility or ongoing transmission when the onsets of invasive GAS cases are separated by long periods.

Given the current limitations of public health surveillance, nursing home staff, especially those responsible for infection control, must be educated specifically about GAS disease and its transmission. Hospital infection control practitioners may be in the best position to find cases and to inform nursing homes when they review culture results for hospital surveillance.

We found further characterization of GAS isolates helpful when confronted with multiple cases of GAS disease in a facility. Even if laboratory resources are scarce, nursing home isolates of GAS should be saved for future testing if additional cases occur. Both e*mm* typing and PFGE are useful tools when attempting to determine whether >2 cases are related. A high percentage of temporally related cases had isolates with indistinguishable PFGE patterns, which suggests that continued transmission of a single strain is occurring in a facility, although reintroduction of a similar strain from the community cannot be excluded. In half of the situations in which a nursing home had 2 invasive cases, additional cases occurred regardless of whether the GAS isolates from the first 2 case-patients had matching PFGE patterns, which suggests a failure of infection control in these facilities. Although knowledge of GAS strain relatedness identified through PFGE or *emm* typing can help identify the source of the GAS infection, circulating within a facility or introduced from the community, we conclude that a thorough investigation is warranted when >1 case has occurred in a facility within a few months. *Emm* typing is not readily available in most public health laboratories; however, the results of *emm* typing of GAS isolates from ongoing ABCs is important for researchers currently developing multivalent GAS vaccines. The types most common among our nursing home case-patients are included in a 26-valent vaccine that has completed a phase II trial (*28*).

Although most invasive GAS disease cases occurring in nursing homes are sporadic, our experience suggests that the time of first awareness of any GAS disease in a nursing home is also the time to assess the extent of spread and institute infection control measures. Clinical syndromes of GAS should be reviewed with staff, and the importance of excluding staff and visitors with illness should be emphasized. Hand hygiene among staff, visitors, and residents needs to be emphasized. We also recommend that surveillance for GAS disease, including noninvasive disease, be implemented and that cultures be obtained from patients with potential cases. If ongoing transmission and disease continue, additional measures, such as performing screening cultures for GAS, can be helpful.
